# *N*-[4-(Benzyl­sulfamo­yl)phen­yl]acetamide

**DOI:** 10.1107/S1600536810027698

**Published:** 2010-07-17

**Authors:** Peter John, Waqar Ahmad, Islam Ullah Khan, Shahzad Sharif, Edward R. T. Tiekink

**Affiliations:** a Materials Chemistry Laboratory, Department of Chemistry, Government College University, Lahore 54000, Pakistan; b Department of Chemistry, University of Malaya, 50603 Kuala Lumpur, Malaysia

## Abstract

A folded conformation is found for the title compound, C_15_H_16_N_2_O_3_S, whereby the benzene rings come into close proximity [centroid–centroid distance = 4.0357 (12) Å and the dihedral angle between them = 24.37 (10)°]. The amide group is coplanar with the benzene ring to which it is bound [C—C—N—C torsion angle = 11.1 (3)°]. In the crystal packing, two-dimensional arrays in the (101) plane are formed *via* N—H⋯O hydrogen bonding.

## Related literature

For background to the pharmacological uses of sulfonamides, see: Beate *et al.* (1998 ▶); Kazmierski *et al.* (2004 ▶). For related structures, see: Khan *et al.* (2010 ▶); Sharif *et al.* (2010 ▶).
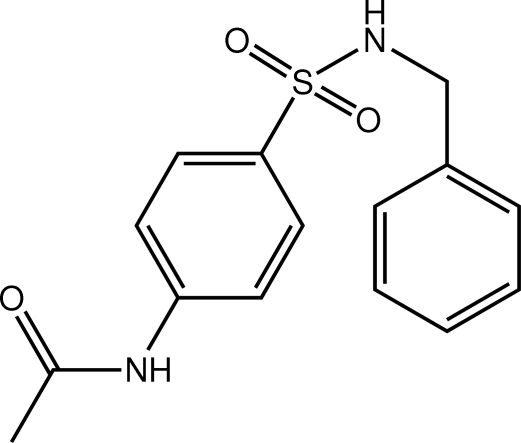


## Experimental

### 

#### Crystal data

C_15_H_16_N_2_O_3_S
*M* *_r_* = 304.37
Monoclinic, 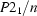

*a* = 9.0646 (9) Å
*b* = 13.6888 (14) Å
*c* = 12.1651 (12) Å
β = 98.635 (5)°
*V* = 1492.4 (3) Å^3^
*Z* = 4
Mo *K*α radiation
μ = 0.23 mm^−1^
*T* = 293 K
0.19 × 0.09 × 0.07 mm

#### Data collection

Bruker APEXII CCD diffractometer
Absorption correction: multi-scan (*SADABS*; Sheldrick, 1996 ▶) *T*_min_ = 0.868, *T*_max_ = 0.948
13777 measured reflections
3577 independent reflections
2689 reflections with *I* > 2σ(*I*)
*R*_int_ = 0.036

#### Refinement

*R*[*F* ^2^ > 2σ(*F* ^2^)] = 0.041
*wR*(*F* ^2^) = 0.124
*S* = 1.02
3577 reflections
197 parameters
2 restraints
H atoms treated by a mixture of independent and constrained refinement
Δρ_max_ = 0.25 e Å^−3^
Δρ_min_ = −0.24 e Å^−3^

### 

Data collection: *APEX2* (Bruker, 2007 ▶); cell refinement: *SAINT* (Bruker, 2007 ▶); data reduction: *SAINT*; program(s) used to solve structure: *SHELXS97* (Sheldrick, 2008 ▶); program(s) used to refine structure: *SHELXL97* (Sheldrick, 2008 ▶); molecular graphics: *ORTEP-3* (Farrugia, 1997 ▶) and *DIAMOND* (Brandenburg, 2006 ▶); software used to prepare material for publication: *publCIF* (Westrip, 2010 ▶).

## Supplementary Material

Crystal structure: contains datablocks global, I. DOI: 10.1107/S1600536810027698/jj2043sup1.cif

Structure factors: contains datablocks I. DOI: 10.1107/S1600536810027698/jj2043Isup2.hkl

Additional supplementary materials:  crystallographic information; 3D view; checkCIF report

## Figures and Tables

**Table 1:** Hydrogen-bond geometry (Å, °)

*D*—H⋯*A*	*D*—H	H⋯*A*	*D*⋯*A*	*D*—H⋯*A*
N1—H1n⋯O3^i^	0.89 (2)	2.00 (2)	2.877 (2)	168 (2)
N2—H2n⋯O2^ii^	0.90 (2)	2.03 (2)	2.921 (2)	172 (2)

Symmetry codes: (i) 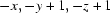; (ii) 
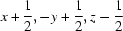
.

**Fig. 1.**
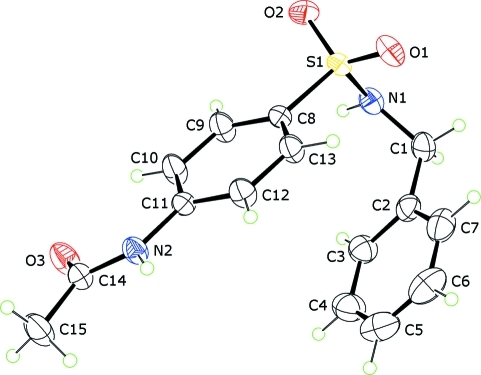
The molecular structure of C15H16N2O3S showing the atom-labelling scheme and displacement ellipsoids at the 35% probability level.

**Fig. 2.**
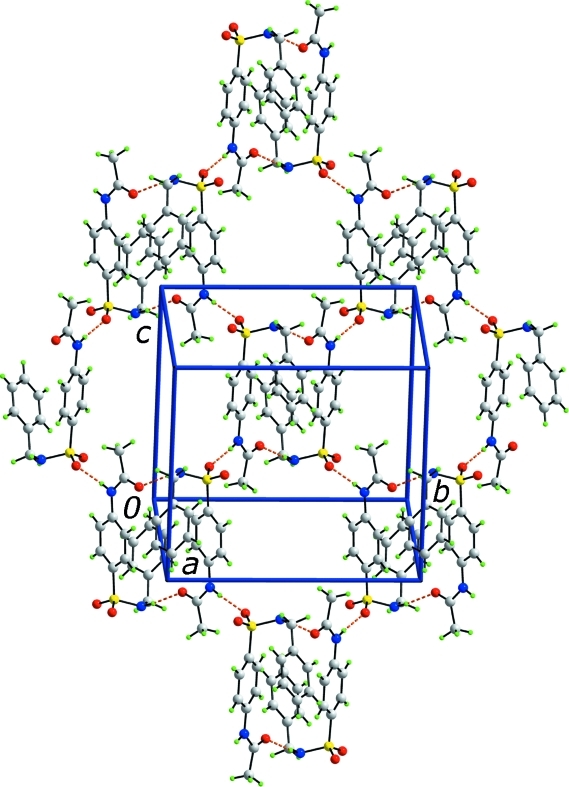
A view of the supramolecular 2-D array in the (1 0 1) plane mediated by N–H···O hydrogen bonding (orange dashed lines) in C15H16N2O3S. Colour code: S, yellow; O, red; N, blue; C, grey; and H, green.

## supplementary crystallographic information

### Comment

Sulfonamide drugs are used, for example, as inhibitors of HIV infection (Kazmierski *et al.*, 2004) and as anti-hypertensive drugs (Beate *et al.*, 1998). In connection with on-going structural studies of sulfonamides (Khan *et al.*, 2010; Sharif *et al.*, 2010), the crystal and molecular structure of the title compound, C_15_H_16_N_2_O_3_S, was investigated.

The molecule of C_15_H_16_N_2_O_3_S has a folded conformation with the benzene ring of the benzyl group somewhat orientated over the S-bound benzene ring. The rings are approximately parallel, forming a dihedral angle of 24.37 (10) °; the distance between the ring centroids is 4.0357 (12) Å. The amide group is essentially co-planar with the ring to which it is bound as seen in the C10–C11–N2–C14 torsion angle of 11.1 (3) °.

The crystal packing is dominated by N–H···O hydrogen bonds whereby the N1–H atom forms a hydrogen bond to the amide-carbonyl, and the amide N2–H forms a contact with the S-bound O2 atom, Table 1. The former leads to centrosymmetric aggregates and these are connected by the latter into a 2-D array in the (1 0 1) plane, Fig. 2.

### Experimental

To 4-acetamidobenzenesulfonyl chloride (498 mg, 2.14 mmol) in distilled water (10 ml) was added benzylamine (234 ml, 2.14 mmol), the reaction mixture was stirred at room temperature while maintaining the pH of the reaction mixture at 8 using 3% sodium carbonate. The progress of the reaction was monitored by TLC. After consumption of all the reactants, the precipitates were filtered, dried and crystallized from methanol to yield colourless crystals.

### Refinement

The C-bound H atoms were geometrically placed (C–H = 0.93–0.97 Å) and refined as riding with *U_iso_*(H) = 1.2–1.5*U_eq_*(C). The N-bound H atom was refined with the distance restraint N–H = 0.88±0.01 Å, and with *U_iso_*(H) = 1.2*U_eq_*(N).

### Crystal data

**Table:**

C_15_H_16_N_2_O_3_S	*F*(000) = 640
*M**_r_* = 304.37	*D*_x_ = 1.355 Mg m^−^^3^
Monoclinic, *P*2_1_/*n*	Mo *K*α radiation, λ = 0.71073 Å
Hall symbol: -P 2yn	Cell parameters from 4154 reflections
*a* = 9.0646 (9) Å	θ = 2.6–27.9°
*b* = 13.6888 (14) Å	µ = 0.23 mm^−^^1^
*c* = 12.1651 (12) Å	*T* = 293 K
β = 98.635 (5)°	Prism, colourless
*V* = 1492.4 (3) Å^3^	0.19 × 0.09 × 0.07 mm
*Z* = 4	

### Data collection

**Table:**

Bruker APEXII CCD diffractometer	3577 independent reflections
Radiation source: fine-focus sealed tube	2689 reflections with *I* > 2σ(*I*)
graphite	*R*_int_ = 0.036
φ and ω scans	θ_max_ = 28.0°, θ_min_ = 2.3°
Absorption correction: multi-scan (*SADABS*; Sheldrick, 1996)	*h* = −11→11
*T*_min_ = 0.868, *T*_max_ = 0.948	*k* = −18→17
13777 measured reflections	*l* = −16→16

### Refinement

**Table:**

Refinement on *F*^2^	Primary atom site location: structure-invariant direct methods
Least-squares matrix: full	Secondary atom site location: difference Fourier map
*R*[*F*^2^ > 2σ(*F*^2^)] = 0.041	Hydrogen site location: inferred from neighbouring sites
*wR*(*F*^2^) = 0.124	H atoms treated by a mixture of independent and constrained refinement
*S* = 1.02	*w* = 1/[σ^2^(*F*_o_^2^) + (0.0643*P*)^2^ + 0.3076*P*] where *P* = (*F*_o_^2^ + 2*F*_c_^2^)/3
3577 reflections	(Δ/σ)_max_ = 0.001
197 parameters	Δρ_max_ = 0.25 e Å^−^^3^
2 restraints	Δρ_min_ = −0.24 e Å^−^^3^

### Special details

**Table:**

Geometry. All s.u.'s (except the s.u. in the dihedral angle between two l.s. planes) are estimated using the full covariance matrix. The cell s.u.'s are taken into account individually in the estimation of s.u.'s in distances, angles and torsion angles; correlations between s.u.'s in cell parameters are only used when they are defined by crystal symmetry. An approximate (isotropic) treatment of cell s.u.'s is used for estimating s.u.'s involving l.s. planes.
Refinement. Refinement of *F*^2^ against ALL reflections. The weighted *R*-factor *wR* and goodness of fit *S* are based on *F*^2^, conventional *R*-factors *R* are based on *F*, with *F* set to zero for negative *F*^2^. The threshold expression of *F*^2^ > 2σ(*F*^2^) is used only for calculating *R*-factors(gt) *etc*. and is not relevant to the choice of reflections for refinement. *R*-factors based on *F*^2^ are statistically about twice as large as those based on *F*, and *R*- factors based on ALL data will be even larger.

### Fractional atomic coordinates and isotropic or equivalent isotropic displacement parameters (Å^2^)

**Table:**

	*x*	*y*	*z*	*U*_iso_*/*U*_eq_	
S1	0.11090 (5)	0.33181 (3)	0.82483 (3)	0.03999 (15)	
O1	0.21873 (16)	0.27094 (10)	0.88809 (10)	0.0536 (4)	
O2	−0.04454 (15)	0.31532 (11)	0.82667 (12)	0.0594 (4)	
O3	0.02478 (16)	0.40834 (11)	0.26180 (11)	0.0596 (4)	
N1	0.14186 (17)	0.44201 (11)	0.86867 (12)	0.0448 (4)	
H1N	0.0786 (18)	0.4846 (12)	0.8311 (15)	0.054*	
N2	0.20419 (16)	0.31175 (10)	0.35277 (11)	0.0399 (3)	
H2N	0.2876 (15)	0.2778 (12)	0.3473 (16)	0.048*	
C1	0.2944 (2)	0.47687 (16)	0.90443 (16)	0.0559 (5)	
H1A	0.3409	0.4347	0.9638	0.067*	
H1B	0.2888	0.5418	0.9355	0.067*	
C2	0.3944 (2)	0.48133 (13)	0.81674 (15)	0.0464 (4)	
C3	0.3606 (2)	0.54229 (14)	0.72574 (17)	0.0532 (5)	
H3	0.2748	0.5805	0.7184	0.064*	
C4	0.4533 (3)	0.54678 (16)	0.6461 (2)	0.0661 (6)	
H4	0.4295	0.5880	0.5852	0.079*	
C5	0.5799 (3)	0.49141 (18)	0.6555 (2)	0.0732 (7)	
H5	0.6416	0.4945	0.6011	0.088*	
C6	0.6154 (3)	0.43158 (18)	0.7452 (2)	0.0745 (7)	
H6	0.7021	0.3943	0.7523	0.089*	
C7	0.5230 (2)	0.42612 (15)	0.8254 (2)	0.0611 (6)	
H7	0.5477	0.3848	0.8860	0.073*	
C8	0.13636 (18)	0.32476 (11)	0.68406 (13)	0.0354 (3)	
C9	0.0308 (2)	0.36444 (16)	0.60359 (15)	0.0499 (5)	
H9	−0.0539	0.3934	0.6238	0.060*	
C10	0.0494 (2)	0.36167 (15)	0.49303 (15)	0.0496 (5)	
H10	−0.0223	0.3888	0.4390	0.059*	
C11	0.17580 (18)	0.31823 (11)	0.46307 (13)	0.0343 (3)	
C12	0.2803 (2)	0.27757 (14)	0.54465 (14)	0.0436 (4)	
H12	0.3647	0.2477	0.5250	0.052*	
C13	0.26102 (19)	0.28078 (13)	0.65483 (14)	0.0426 (4)	
H13	0.3321	0.2533	0.7091	0.051*	
C14	0.1335 (2)	0.35627 (13)	0.26110 (14)	0.0418 (4)	
C15	0.1978 (3)	0.33645 (17)	0.15660 (16)	0.0605 (6)	
H15A	0.1635	0.2741	0.1270	0.091*	
H15B	0.3048	0.3360	0.1730	0.091*	
H15C	0.1664	0.3865	0.1029	0.091*	

### Atomic displacement parameters (Å^2^)

**Table:**

	*U*^11^	*U*^22^	*U*^33^	*U*^12^	*U*^13^	*U*^23^
S1	0.0444 (3)	0.0476 (3)	0.0295 (2)	−0.00397 (18)	0.01067 (17)	0.00348 (17)
O1	0.0718 (10)	0.0527 (7)	0.0358 (6)	0.0085 (6)	0.0066 (6)	0.0080 (6)
O2	0.0492 (8)	0.0830 (10)	0.0502 (8)	−0.0168 (7)	0.0209 (6)	0.0030 (7)
O3	0.0599 (9)	0.0774 (10)	0.0420 (7)	0.0218 (7)	0.0096 (6)	0.0121 (7)
N1	0.0510 (9)	0.0497 (9)	0.0341 (7)	0.0033 (7)	0.0079 (6)	−0.0005 (6)
N2	0.0407 (8)	0.0491 (8)	0.0312 (7)	0.0066 (6)	0.0094 (6)	−0.0011 (6)
C1	0.0667 (13)	0.0620 (12)	0.0363 (9)	−0.0135 (10)	−0.0015 (9)	−0.0055 (9)
C2	0.0470 (10)	0.0429 (9)	0.0467 (10)	−0.0085 (8)	−0.0014 (8)	−0.0028 (8)
C3	0.0547 (12)	0.0492 (10)	0.0555 (11)	0.0010 (8)	0.0081 (9)	0.0034 (9)
C4	0.0772 (16)	0.0596 (13)	0.0634 (14)	−0.0104 (11)	0.0167 (12)	0.0071 (11)
C5	0.0672 (15)	0.0670 (14)	0.0917 (18)	−0.0175 (12)	0.0319 (14)	−0.0142 (13)
C6	0.0467 (13)	0.0666 (14)	0.109 (2)	−0.0009 (10)	0.0096 (13)	−0.0133 (14)
C7	0.0554 (13)	0.0516 (11)	0.0706 (14)	−0.0016 (9)	−0.0091 (11)	0.0039 (10)
C8	0.0362 (9)	0.0418 (8)	0.0290 (7)	−0.0043 (6)	0.0079 (6)	0.0004 (6)
C9	0.0395 (10)	0.0759 (13)	0.0357 (9)	0.0162 (9)	0.0105 (7)	0.0016 (9)
C10	0.0420 (10)	0.0736 (12)	0.0329 (9)	0.0161 (9)	0.0050 (7)	0.0031 (9)
C11	0.0351 (8)	0.0379 (8)	0.0305 (7)	−0.0024 (6)	0.0074 (6)	−0.0015 (6)
C12	0.0397 (9)	0.0541 (10)	0.0383 (9)	0.0122 (8)	0.0098 (7)	0.0001 (8)
C13	0.0408 (10)	0.0523 (10)	0.0341 (8)	0.0080 (8)	0.0038 (7)	0.0044 (7)
C14	0.0461 (10)	0.0468 (9)	0.0327 (8)	−0.0030 (8)	0.0063 (7)	−0.0006 (7)
C15	0.0711 (14)	0.0797 (15)	0.0328 (9)	0.0057 (11)	0.0145 (9)	0.0024 (9)

### Geometric parameters (Å, °)

**Table:**

S1—O1	1.4199 (13)	C5—C6	1.363 (4)
S1—O2	1.4304 (14)	C5—H5	0.9300
S1—N1	1.6105 (16)	C6—C7	1.380 (3)
S1—C8	1.7647 (16)	C6—H6	0.9300
O3—C14	1.217 (2)	C7—H7	0.9300
N1—C1	1.466 (2)	C8—C9	1.374 (2)
N1—H1N	0.893 (9)	C8—C13	1.373 (2)
N2—C14	1.346 (2)	C9—C10	1.381 (2)
N2—C11	1.4064 (19)	C9—H9	0.9300
N2—H2N	0.899 (9)	C10—C11	1.387 (2)
C1—C2	1.501 (3)	C10—H10	0.9300
C1—H1A	0.9700	C11—C12	1.382 (2)
C1—H1B	0.9700	C12—C13	1.378 (2)
C2—C7	1.380 (3)	C12—H12	0.9300
C2—C3	1.383 (3)	C13—H13	0.9300
C3—C4	1.376 (3)	C14—C15	1.501 (2)
C3—H3	0.9300	C15—H15A	0.9600
C4—C5	1.366 (3)	C15—H15B	0.9600
C4—H4	0.9300	C15—H15C	0.9600
			
O1—S1—O2	119.90 (9)	C7—C6—H6	119.9
O1—S1—N1	107.38 (8)	C2—C7—C6	120.8 (2)
O2—S1—N1	105.41 (9)	C2—C7—H7	119.6
O1—S1—C8	108.31 (8)	C6—C7—H7	119.6
O2—S1—C8	106.21 (8)	C9—C8—C13	120.05 (15)
N1—S1—C8	109.33 (8)	C9—C8—S1	119.43 (13)
C1—N1—S1	120.86 (14)	C13—C8—S1	120.52 (13)
C1—N1—H1N	116.3 (13)	C8—C9—C10	120.62 (16)
S1—N1—H1N	112.0 (13)	C8—C9—H9	119.7
C14—N2—C11	129.01 (14)	C10—C9—H9	119.7
C14—N2—H2N	118.3 (13)	C9—C10—C11	119.62 (16)
C11—N2—H2N	112.4 (13)	C9—C10—H10	120.2
N1—C1—C2	116.46 (15)	C11—C10—H10	120.2
N1—C1—H1A	108.2	C12—C11—C10	119.21 (15)
C2—C1—H1A	108.2	C12—C11—N2	117.12 (14)
N1—C1—H1B	108.2	C10—C11—N2	123.66 (15)
C2—C1—H1B	108.2	C13—C12—C11	120.82 (15)
H1A—C1—H1B	107.3	C13—C12—H12	119.6
C7—C2—C3	118.3 (2)	C11—C12—H12	119.6
C7—C2—C1	121.10 (19)	C8—C13—C12	119.68 (16)
C3—C2—C1	120.57 (18)	C8—C13—H13	120.2
C4—C3—C2	120.4 (2)	C12—C13—H13	120.2
C4—C3—H3	119.8	O3—C14—N2	123.00 (16)
C2—C3—H3	119.8	O3—C14—C15	121.99 (17)
C5—C4—C3	120.7 (2)	N2—C14—C15	115.01 (16)
C5—C4—H4	119.7	C14—C15—H15A	109.5
C3—C4—H4	119.7	C14—C15—H15B	109.5
C6—C5—C4	119.7 (2)	H15A—C15—H15B	109.5
C6—C5—H5	120.2	C14—C15—H15C	109.5
C4—C5—H5	120.2	H15A—C15—H15C	109.5
C5—C6—C7	120.2 (2)	H15B—C15—H15C	109.5
C5—C6—H6	119.9		
			
O1—S1—N1—C1	35.74 (15)	O1—S1—C8—C13	−11.08 (17)
O2—S1—N1—C1	164.65 (14)	O2—S1—C8—C13	−141.10 (15)
C8—S1—N1—C1	−81.56 (15)	N1—S1—C8—C13	105.63 (15)
S1—N1—C1—C2	64.4 (2)	C13—C8—C9—C10	−0.8 (3)
N1—C1—C2—C7	−118.2 (2)	S1—C8—C9—C10	178.69 (16)
N1—C1—C2—C3	62.7 (2)	C8—C9—C10—C11	0.1 (3)
C7—C2—C3—C4	0.4 (3)	C9—C10—C11—C12	0.6 (3)
C1—C2—C3—C4	179.41 (19)	C9—C10—C11—N2	179.85 (18)
C2—C3—C4—C5	−0.1 (3)	C14—N2—C11—C12	−169.65 (17)
C3—C4—C5—C6	−0.5 (4)	C14—N2—C11—C10	11.1 (3)
C4—C5—C6—C7	0.7 (4)	C10—C11—C12—C13	−0.8 (3)
C3—C2—C7—C6	−0.1 (3)	N2—C11—C12—C13	179.98 (16)
C1—C2—C7—C6	−179.14 (19)	C9—C8—C13—C12	0.6 (3)
C5—C6—C7—C2	−0.5 (3)	S1—C8—C13—C12	−178.81 (14)
O1—S1—C8—C9	169.47 (15)	C11—C12—C13—C8	0.1 (3)
O2—S1—C8—C9	39.45 (17)	C11—N2—C14—O3	−3.2 (3)
N1—S1—C8—C9	−73.82 (16)	C11—N2—C14—C15	177.27 (17)

### Hydrogen-bond geometry (Å, °)

**Table:**

*D*—H···*A*	*D*—H	H···*A*	*D*···*A*	*D*—H···*A*
N1—H1n···O3^i^	0.894 (17)	1.996 (17)	2.877 (2)	168.2 (16)
N2—H2n···O2^ii^	0.898 (15)	2.029 (15)	2.921 (2)	171.5 (16)

Symmetry codes: (i) −*x*, −*y*+1, −*z*+1; (ii) *x*+1/2, −*y*+1/2, *z*−1/2.

## References

[bb1] Beate, G., Nadenik, P. & Wagner, H. (1998). WO Patent No. 9855481.

[bb2] Brandenburg, K. (2006). *DIAMOND* Crystal Impact GbR, Bonn, Germany.

[bb3] Bruker (2007). *APEX2* and *SAINT* Bruker AXS Inc., Madison Wisconsin, USA.

[bb4] Farrugia, L. J. (1997). *J. Appl. Cryst.* **30**, 565.

[bb5] Kazmierski, W. M., Aquino, C. J., Bifulco, N., Boros, E. E., Chauder, B. A., Chong, P. Y., Duan, M., Deanda, F. Jr, Koble, C. S., Mclean, E. W., Peckham, J. P., Perkins, A. C., Thompson, J. B. & Vanderwall, D. (2004). WO Patent No. 2004054974.

[bb6] Khan, I. U., Mariam, I., Zia-ur-Rehman, M., Arif Sajjad, M. & Sharif, S. (2010). *Acta Cryst.* E**66**, o1088. 10.1107/S160053681001322X PMC2979209 21579141

[bb7] Sharif, S., Iqbal, H., Khan, I. U., John, P. & Tiekink, E. R. T. (2010). *Acta Cryst.* E**66**, o1288. 10.1107/S1600536810016119 PMC2979509 21579386

[bb8] Sheldrick, G. M. (1996). *SADABS* University of Göttingen, Germany.

[bb9] Sheldrick, G. M. (2008). *Acta Cryst.* A**64**, 112–122. 10.1107/S0108767307043930 18156677

[bb10] Westrip, S. P. (2010). *J. Appl. Cryst.* **43**, 920–925.

